# Current State and Future Directions of Diagnostic Imaging and Interpretation in Pediatric Radiology

**DOI:** 10.3390/diagnostics16071007

**Published:** 2026-03-27

**Authors:** André Lollert

**Affiliations:** Department of Diagnostic and Interventional Radiology, Section of Pediatric Radiology, Medical Center of the Johannes Gutenberg-University, 55131 Mainz, Germany; andre.lollert@unimedizin-mainz.de

Pediatric radiology is an inherently technology-dependent medical subspecialty [1]. In the past decades, medical imaging procedures in children increased substantially with the exception of a stable or decreasing number of computed tomography (CT) examinations, due to radiation protection efforts [2,3]. However, there is a significant lack of specialized, board-certified pediatric radiologists to cover the unique imaging requirements in children. The wide patient stature range from premature infants to potentially obese adolescents underlines the need for specific imaging expertise. For example, higher radiation doses have been reported for CT examinations of almost all body regions if children were examined in non-pediatric facilities [4]. In addition, reporting of pediatric imaging procedure results by adult/general radiologists or even non-radiology specialists (in particular during off-duty hours) might lead to wrong or delayed diagnoses [5,6,7]. Furthermore, novel techniques are developed for (and validated in) adult patient populations in the majority of cases. This means that they have to be adapted prior to their use in children and adolescents [8]. These facts imply the need for advancements in pediatric radiology to ensure optimal care for the smallest of our patients. If new scientific inputs emerge into clinical practice, we might be able to compensate at least partially for the lack of specialists.

A few years ago, we summarized modern developments in pediatric radiology, concentrating on CT dose reduction, advances in magnetic resonance imaging (MRI) techniques, and hybrid imaging such as positron emission tomography (PET)/MRI [9]. Nowadays, the clinical introduction of the photon-counting CT technology has emerged as a major step towards image quality optimization, for example, by applying multi-energy and fast-pitch scanning modes, virtual mono-energetic image reconstructions, or ultra-high resolution scans [10]. In addition, it offers a large potential for dose reduction, especially in pediatric patients [11]. It has been applied to different disorders, including, but not limited to, cardiovascular disease [12,13], lung pathologies [14], neurological disorders [15], and abdominopelvic pathologies [16]. Thus, photon-counting CT marks a major technological advancement in CT imaging for pediatric patients, as it can improve image quality while reducing the radiation dose and/or amount of necessary contrast media [10].

A major contemporary challenge in almost all medical subspecialties, including pediatric radiology, is the evidence-based implementation of techniques using artificial intelligence (AI) [17]. As mentioned above, adult-trained deep learning models do not necessarily transfer to pediatric patient collectives without adaptations [18]. Still, these techniques are already used for different applications, which can be assorted to different groups. First, AI tools can assist the radiologist in making a diagnosis. Examples include bone age estimation [19] or fracture assessment [20]. Furthermore, AI can be used to optimize workflow, for example, by performing automated tumor segmentations [21], which would be a time-consuming task if performed manually by the radiologist. Finally, AI is already implemented into clinical routines as a tool to enhance image quality by reducing artifacts due to an acceleration of MRI pulse sequences [22]. Furthermore, AI might allow for a reduction in the amount of necessary contrast media [23] or PET tracers [24], which consequently lowers potential side effects or ionizing radiation.

While most medical deep learning models are specifically developed to aid in diagnostics of a disorder or for automated image analysis, other approaches aim to use publicly available AI tools such as ChatGPT, e.g., for pediatric fracture evaluation [25] or bone age estimation [26]. However, these approaches have to be followed with care, as recently demonstrated in a study showing a low sensitivity when using ChatGPT for the detection of subtle pneumothoraces in pediatric patients [27]. This implies that a cautious evaluation of new AI methods is necessary prior to their implementation into daily clinical routines.

Recent advances in the field of ultrasound as a point-of-care imaging modality head for improvements in diagnostics by adding quantitative information. For example, radiomics-based analyses of kidney sonograms might differentiate between acute and chronic renal disorders [28]. Another example of a quantitative parameter is organ stiffness measured by ultrasound elastography. This parameter might be exemplarily used to differentiate between different types of spleen pathology in children [29]. Both of the aforementioned examples underline the strive for the acquisition of parameters that exceed pure visual imaging analysis in order to enhance diagnostic possibilities.

While new techniques, as described above, have to be developed and implemented continuously, the standardization of established methods also remains an ongoing challenge in pediatric radiology. First, this applies to the methodological aspect of how image acquisition is practically performed and by whom. Exemplarily, for cross-sectional imaging of neonates and infants, potentially requiring anesthesia, alternative methods such as “feed-and-wrap” approaches [30] are still inconsistently applied [31]. Therefore, retrospective data analyses concerning the use of clinically well-established methods might generate scientific evidence for their utilization. Second, harmonization of imaging protocols, for example, MRI sequences and sequence parameters, among institutions worldwide should be a topic to advance on [32]. If imaging is harmonized across specialized pediatric radiology departments, it may be more readily transferable to hospitals or regions where dedicated pediatric radiologists are unavailable.

In summary, pediatric radiologists will have to stay at the front of new technological developments to be able to provide high-quality care for children and adolescents. Collecting new evidence concerning diagnostics of pediatric diseases by imaging will help to transfer scientific research into clinical practice.

## Abbreviations

The following abbreviations are used in this manuscript:

AI	Artificial Intelligence
CT	Computed Tomography
MRI	Magnetic Resonance Imaging
PET	Positron Emission Tomography
